# Distinct maternal DNA methylation associations with gestational age at early and late-mid term pregnancy in a low- and middle-income country: evaluation of biological, genetic, and psychosocial contributors

**DOI:** 10.1186/s12884-025-08037-6

**Published:** 2025-10-10

**Authors:** Marcia Smiti Jude, Chaini Konwar, Robyn J. McQuaid, Farooq Ghani, Nazneen Islam, Sharifa Lalani, Sarah M. Merrill, Fizza Fatima, Julia L. MacIsaac, Ntonghanwah Forcheh, Calen P. Ryan, Nanette R. Lee, Christopher W. Kuzawa, Michael S. Kobor, Shahirose Sadrudin Premji, Maternal-infant Global Health Team (MiGHT) – Collaborators in Research, Neelofur Babar, Aliyah Dosani, Imtiaz Jehan, Nicole Letourneau, Mohamoud Merali, Ayesha Mian, Joseph Wangira Musana, Christopher T. Naugler, Sidrah Nausheen, Christine Okoko, Geoffrey Omuse, Saima Sachwani, Pauline Samia, Kiran Shaikh, Rozina Shazad, Salima Sulaiman, Sikolia Wanyonyi, Ilona S. Yim

**Affiliations:** 1 BC Children’s Hospital Research Institute (BCCHR), 950 West 28th Avenue, Vancouver, BC Canada; 2 Department of Medical Genetics, Faculty of Medicine, University of British Columbia, Vancouver, BC Canada; 3 Department of Neuroscience, Carleton University, Ottawa, ON Canada; 4 University of Ottawa Institute of Mental Health Research, Ottawa, ON Canada; 5 Department of Pathology and Microbiology, Aga Khan University, Karachi, Sindh Pakistan; 6 Molecular Pathology, Clinical Laboratory Medicine, Aga Khan University Hospital, Karachi, Sindh Pakistan; 7 School of Nursing and Midwifery, Aga Khan University, Karachi, Sindh Pakistan; 8 Department of Psychiatry and Human Behavior, The Warren Alpert Medical School at Brown University, Providence, Rhode Island, USA; 9 School of Nursing, Faculty of Health Sciences, Queen’s University, Kingston, ON Canada; 10 Robert N. Butler Columbia Aging Center, Mailman School of Public Health, Columbia University, New York, NY USA; 11 Office of Population Studies, University of San Carlos, Talamban, Cebu City, Philippines; 12 Department of Human Evolutionary Biology, Harvard University, Cambridge, MA USA; 13 Edwin S. H. Leong Centre for Healthy Aging, Faculty of Medicine, University of British Columbia, Vancouver, BC Canada; 14 Sally Smith Chair in Nursing, School of Nursing, Faculty of Health Sciences, Queen’s University, Kingston, ON Canada

**Keywords:** Gestational age at birth, Pregnancy, DNA methylation, Maternal blood, Fetal environment, LMIC, Immune cell types, MiGHT

## Abstract

Mothers undergo physiological and molecular changes over the course of gestation. These modifications “get under the skin” and may be reflected in the maternal epigenome through processes such as DNA methylation. Such an epigenetic mark may offer insights into maternal responses to prenatal influences and biological cues from the developing fetus, thereby functioning as an indirect indicator of the conditions the fetus experiences in utero. We measured whole blood DNA methylation using the MethylationEPIC BeadChip Infinium microarray v1.0 in 22 pregnant women from Pakistan, a low- and middle-income country (LMIC), at two timepoints during their term pregnancies (early: 10–19 weeks and late-mid: 22–29 weeks). We used DNA methylation profiles to predict immune cell proportions and tested differences in these proportions and DNA methylation patterns between the two timepoints. Further, we evaluated DNA methylation associations with gestational age at each timepoint and examined the contribution of genetic, psychosocial, and biological factors. Our analyses documented changes in immune cell proportions and DNA methylation profiles over the course of gestation, albeit in a small percentage of the measured DNA methylome. We also observed timepoint-specific DNA methylation associations with gestational age, predominantly at early pregnancy, with predicted interleukin-6 level and socioeconomic status contributing to a few of these associations. On comparing to three external cohorts from different sociocultural contexts, we also noted these signatures to be unique to LMIC settings. Overall, these changes measured in term pregnancies may be used to assess both fluctuations in pregnancy and birth outcomes, particular in women from LMIC settings.

**Supplementary Information:**

The online version contains supplementary material available at 10.1186/s12884-025-08037-6.

## Background

Pregnancy is a period of dynamic changes occurring in the mother to support the fetus [1] and maintain maternal health. These changes vary across trimesters, reflecting the evolving demands of pregnancy. In the early stages, the emphasis is on the establishment and maintenance of pregnancy, including tolerance to the fetus, while the later stages are focused on optimizing nutrient and oxygen delivery to the rapidly growing fetus, along with preparing for labor and delivery [1–4]. Understanding normative changes in term pregnancies across gestation is vital in establishing a framework to assess fluctuations over the course of pregnancy, particularly in low- and middle-income countries (LMIC). Socioeconomic disadvantages experienced by pregnant women from LMIC pose challenges to the wellbeing of both the mother and child. There is an increased prevalence of physiological and psychological health problems, and poverty associated distress experienced by these women which influences fetal developmental timeline [5]. The cumulative influence of these adverse factors necessitates perinatal research in specific LMIC contexts to improve maternal and child health outcomes in these settings.

Immune and molecular changes occur throughout gestation to enable successful maintenance of the pregnancy [2]. Deviations from a tightly regulated immune schedule and disturbances in molecular processes impact the programming of maternal adaptations over the course of gestation and thus, result in adverse pregnancy outcomes [6]. These regulated immune changes can be elucidated by the specific immune cell profiles corresponding to each trimester in healthy pregnant women ^3^. Attenuated adaptive immune responses over the course of pregnancy have been noted [7], with decreased T cell (CD4 + T and CD8 + T) and natural killer (NK) cell proportions and function in the second trimester, as well as low circulating CD19 + B cell proportions in the third trimester. On the other hand, there is an expansion of innate immune cell proportions during pregnancy, with increased neutrophils and monocytes at late-mid pregnancy [7]. These established cellular changes to the immune landscape that occur during pregnancy are also reflected in epigenetic marks such as DNA methylation, a commonly investigated chemical modification in human population studies [8–10]. Epigenetic investigations in healthy pregnant women comparing different timepoints in gestation have identified cell type independent DNA methylation signatures in processes linked to early organogenesis, immune activity, metabolic processes, and cell cycle and solute transport functions, among others [10]. These molecular changes thereby represent continual adjustments of the maternal physiological milieu to accommodate the growing fetus during gestation.

Fetal developmental trajectories are closely linked to gestational age. Slight variations in gestational age at birth, even within the normative window of a term pregnancy (> 37 weeks gestation), influence at-birth characteristics and later health outcomes [11]. Specifically, infants born at early term are more likely to develop learning and physical difficulties such as poor vision and hearing impairments, as well as increased risk of emergency hospital admissions during early childhood compared to infants who are born closer to full term [12–14]. Gestational age has also been associated with molecular marks such as DNA methylation in birth tissues [15–21]. Epigenetic investigations of pregnant women, particularly over the course of pregnancy, as it links to gestational age at birth are sparse. Understanding these dynamic molecular patterns in pregnant women will aid in making informed healthcare decisions thereby improving birth outcomes.

The biological underpinnings of the association between DNA methylation and gestational age are poorly understood. Although the contribution of cell type proportions to this association was recently investigated in cord blood [20], the influence of maternal experiences during pregnancy, such as prenatal stress, on gestational age specific differential DNA methylation needs to be more deeply elucidated. The psychological state of women may be labile and particularly sensitive to stressful experiences during pregnancy, and together with departures from normal physiological responses, can affect the length of gestation [22, 23]. Specifically, increases in pregnancy-related anxiety and perceived stress from second to third trimester have been associated with shortened gestation and preterm birth [24, 25]. Further, socioeconomic stressors, including the circumstances and contexts of individuals’ lives as well as other social determinants of health, vary across pregnancy and might have implications for subsequent pregnancy outcomes [26, 27]. Such investigations of the psychosocial complexities of pregnancy, however, have been conducted predominantly in populations belonging to high-income countries such as the United States of America [24], Europe [28], and Canada [25], and their generalizability beyond these settings, such as in the large populations from low- and middle-income countries (LMIC), is unclear. Taking these varying contexts into account, continual adjustments of multiple physiological systems such as neuroendocrine, metabolic, and cardiovascular systems, are initiated in response to these prenatal stressors with an aim to achieve homeostasis [29]. Biomarkers released because of the stress experienced, indicators of ‘wear and tear’ on the brain and physiological systems during pregnancy, together with blood pressure measures, have been combined to form the allostatic load index, and studied as a predictor of gestational age at birth [30, 31]. While these individual factors have been independently assessed for their potential contributions to DNA methylation variance [32–35], their roles in explaining maternal DNA methylation effects related to gestational age have not yet been examined to our knowledge.

Socioeconomic and cultural conditions surrounding pregnancy and childbirth in high-income populations differ significantly from those in LMIC. Owing to these differences and the additional challenges in directly studying the fetus in utero, our overarching hypothesis was to examine if the maternal epigenome, a proxy for the in utero developmental environment of the fetus, was predictive of gestational age at birth, in an LMIC population. We further hypothesized distinct gestational age specific maternal DNA methylation signatures at two timepoints during pregnancy and further expected these signatures to be unique to LMIC settings. To test this, we recruited pregnant women from Sindh, Pakistan in a longitudinal manner and investigated two timepoints in their gestation: early (10–19 weeks) and late-mid (22–29 weeks) pregnancy. Next, we assembled three external cohorts representing different socioeconomic and cultural settings to evaluate how distinct our LMIC-specific findings were. Further, we examined the influence of genetic, psychosocial, and biological factors, including immune system function as operationalized through DNA methylation-derived estimated inflammatory marker interluekin-6 (IL-6), on the noted maternal DNA methylation patterns in predicting gestational age. Utilizing available matched maternal blood data, we compared immune cell type and DNA methylation profiles between the two pregnancy timepoints, to corroborate our findings with published results of molecular and cell type changes over the course of gestation.

## Methods

### Cohort description

The cohort of 40 pregnant women in the current study is a representative sub-sample drawn from a large cohort comprising 1861 women recruited from Sindh, Pakistan [36]. For these 40 women, matched venous blood was collected during two timepoints in their pregnancy – (i) early pregnancy (10–19 weeks gestation) and (ii) late-mid pregnancy (22–29 weeks gestation) with at least 10 weeks between measures, along with complete psychosocial assessments at both timepoints. Participants were included in the study based on criteria described in the Supplementary Methods (Additional file 2). During sample quality checks, only matched samples from 26 women were retained, while the remaining were dropped from the current study, as described later in Methods. None of these 26 women reported any known health conditions. Among the 26 pregnant women, four women had preterm deliveries (gestational age < 37 weeks) and were thus excluded from the epigenome-wide association studies (EWAS), resulting in a final sample size of 22 women who carried their pregnancy to term (> 37 weeks’ gestation). However, these four preterm women were used in a post-hoc analysis to evaluate whether DNA methylation signatures identified in term were also altered in preterm pregnancies. Table 1 provides a detailed description of the cohort demographics (n = 22) obtained through mothers’ self-reports and hospital records. All research was performed in accordance with relevant guidelines and regulations after ethics approval received from: National Bioethics Committee of Pakistan [No.4–87/NBC-286-Y2], Aga Khan University Ethics Review Committee, Karachi, Sindh, Pakistan [5003-SON-ERC-17], Queen’s University Health Sciences & Affiliated Teaching Hospitals Research Ethics Board [NURS-566–23], University of Calgary Conjoint Health Research Ethics Board [REB17-1148_REN5], York University Office of Research Ethics [2018–184], and Mount Royal University Human Research Ethics Board [File ID#10116]. Written informed consent was obtained from the participants.

**Table 1:** Demographic characteristics of the study participants based on self-reports and hospital records

Variable	Level	Overall n (%)
n		22
Sample collection site	Garden	3 (13.64)
	Karimabad	8 (36.36)
	Kharadar	6 (27.27)
	Hyderabad	5 (22.73)
Age at recruitment (years) *		26.09 (4.05)
Gestational age (weeks) *		38.52 (1.12)
Self-reported ethnicity	Memon	2 (9.1)
	Sindhi	4 (18.2)
	Katchi	1 (4.5)
	Punjabi	3 (13.6)
	Balochi	2 (9.1)
	Urdu-Mahajir	8 (36.4)
	Other ^φ^	2 (9.1)
Gross household income	Rs 10,001- 20,000	6 (27.3)
	Rs 20,001- 40,000	3 (13.6)
	More than Rs 40,000	12 (54.5)
	Don’t know/Prefer not to say	1 (4.5)
Occupation	Non-government employee	2 (9.09)
	Student	1 (4.55)
	Homemaker	19 (86.36)
Education	Primary school completed	1 (4.55)
	Secondary/High school completed	4 (18.18)
	College/University completed	11 (50.00)
	Post graduate degree	6 (27.27)

The values in the last column are given as mean (standard deviation) for the numerical variables (indicated with *), and number (percentage) for the categorical variables

^Φ^ Other includes Chitrali, Khuwar, Urdu and Hinko

### DNA extraction and quantification

Whole blood was collected into EDTA-coated tubes at two timepoints in pregnancy, and DNA extraction was performed using QIAsymphony DSP DNA mini kit, Qiagen. Quality and quantity of DNA was assessed using NanoDrop spectrophotometry. DNA was stored at −80 C in ultra-low freezer for subsequent assays. For DNA methylation quantification, 750ng of genomic DNA was extracted and bisulfite converted using EZ DNA Methylation Kit (Zymo Research). The samples were run in one batch according to the manufacturer's protocol (Illumina). DNA methylation on these maternal blood samples was quantified using the Illumina Infinium MethylationEPIC BeadChip platform (850K array) that measures 866,895 CpGs on the genome. Raw IDATS obtained after scanning the chips by a iScan 2000 (Illumina) were read into R and the *minfi* R package was used to obtain DNA methylation levels at each CpG (β value ranging from 0–1) [37]. Subsequent data preprocessing and analyses were done in R (version 4.0.3).

Maternal blood samples were collected at two timepoints, therefore, longitudinal sample identity checks were performed using 59 SNP probes in the 850 K array through unsupervised hierarchical clustering, and functions from the *ewastools* R package [38]. These checks were based on the premise that samples from the same individual have similar genetic fingerprints and therefore should have highly correlated β values. We noted that 26 of the 40 samples measured at one timepoint clustered together with the corresponding samples at the second timepoint, and these sample pairs were therefore included in subsequent analyses. Additional sample quality checks were performed, as described previously [39, 40]. We carried out probe filtering [41], functional normalization to correct for probe type differences, and batch correction using the ComBat function in the *sva R package* to account for chip and row effects [42]. To identify informative CpGs in maternal blood during pregnancy and reduce data dimensionality, we applied a two-step variability filter. We first excluded CpGs previously reported as non-variable in whole blood (n = 114,204) and then selected for CpGs with an interquantile range (90th–10th percentile) of beta values ≥ 5% across our 22 samples [43]. Overall, 224,663 variable and good quality probes were retained for statistical analyses.

### Allostatic load measure

Six biomarkers, reflecting stress related multisystem dysregulation (up or down regulation), including cortisol (ug/dl), glycosylated hemoglobin (whole blood HbA1 (%)), C-reactive protein (mg/dl), total cholesterol (mg/dl), and systolic and diastolic blood pressure (mmHg)) were measured. Details on the six biomarkers are provided in Additional file 1- Supplementary Table 1. Cortisol was measured between 11am and 2pm to control for diurnal variation and was adjusted for time of day (minutes) and the gestational week calculated from the last menstrual period using multiple regression analysis. As both positive and negative deviation of cortisol levels from norm represent dysregulation, the predicted cortisol was then subtracted from the actual cortisol value with the overall mean added to this difference. Allostatic load was then estimated by averaging the standardized scores for each of the six mediators (mean = 0 and variance = 1) [44].

### Pregnancy psychosocial measures

Pregnant mothers completed relevant questionnaires corresponding to the two timepoints (early: 10–19 weeks gestation, late-mid pregnancy: 22–29 weeks gestation) at the time of maternal blood collection. Specifically, psychosocial measures including maternal prenatal mental health (pregnancy-related anxiety, depression), and socioeconomic status indices (once, only at the early timepoint) were collected. Depending on preference and/or literacy women either completed a self-report questionnaire or a trained interviewer elicited a response and recorded it on the questionnaire. Details on these measures are provided in the Supplementary Methods (Additional files 1 and 2).

### DNA methylation-based interleukin-6 score prediction

DNA methylation-based IL-6 predictor was leveraged to obtain proxy interleukin-6 (IL-6) scores, as this pro-inflammatory cytokine is released in response to stressful stimuli and important in immune function [45]. Proxy IL-6 scores at the two pregnancy timepoints were calculated as a weighted average of 35 predictive CpGs that were previously found to accurately predict serum IL-6 levels. On multiplying the coefficient weights for these previously identified 35 CpGs and their corresponding beta values in the two timepoints and taking an average, we calculated proxy IL-6 scores. Only 26 of the 35 predictive CpGs were available in our dataset after data preprocessing, and thus only these available predictive CpGs were used to calculate the proxy IL-6 scores. The IL-6 scores estimated at the two timepoints using this DNA methylation-based predictor exhibited a modest correlation of 0.44 (Additional file 2-Supplementary Fig. 1).

### DNA methylation-based estimation of immune cell proportions

Cellular composition is a key contributor to whole blood DNA methylation variation [46, 47]. In the absence of complete cell count data for the study samples, the extended IDOL (Identifying Optimal DNA methylation Libraries) reference was used to estimate twelve leukocyte subtypes, namely neutrophils, eosinophils, basophils, monocytes, naïve and memory B cells, naïve and memory CD4 + and CD8 + T cells, natural killer (NK), and T regulatory cells [48]. Using pairwise t-tests and applying a Bonferroni multiple test correction [49], we evaluated significant differences in proportions of individual cell types between early and late-mid pregnancy timepoints. Effect sizes were classified as ‘small’ (d = 0.2–0.49), ‘medium’ (d = 0.5–0.79), and ‘large’ (d = ≥ 0.8) based on benchmarks suggested by Cohen [50].

To identify gestational age specific DNA methylation changes that are independent of shifts in cell type composition during pregnancy, we performed a principal component analysis on the estimated cell type proportions and included principal components (PCs) as covariates to adjust for technical noise associated with individual variation in immune cell profiles. Using the pcaCoDa() function in the *robCompositions* R package, isometric logratio (ilr) transformation and robust principal component analysis were performed on the compositional cell-type data for each timepoint [51]. The first two PCs, representing > 95% of the overall variation among the 12 estimated cell types across individuals, were included as covariates in the statistical models in Methods, Sect. 2.8.

### Maternal DNA methylation changes between early and late-mid pregnancy timepoints

To identify molecular patterns that change over the course of pregnancy, we compared DNA methylation levels at 224,663 CpGs between the early and late-mid pregnancy timepoints in the matched cohort of 22 pregnant women. We applied robust linear mixed models with CpG beta values at both early and late-mid pregnancy as outcome, with individual as a random effect using the *robustlmm* R package [52]. Maternal age and five out of the 12 estimated cell types that were significantly different between the investigated timepoints were included as covariates in the model. For these five cell types, a difference in estimated proportions between early and late-mid pregnancy was used.

$$\begin{aligned} &CpG\;\beta\;value\sim Timepoint+Maternal\;age\\&+\Delta\text{Bmem}+\Delta\text{CD}4\text{mem}+\Delta\text{CD}8\text{mem}\\&+\Delta\text{Neu}+\Delta\text{NK}+\left(1\mid Individual\right)\end{aligned}$$

*p*-values were calculated using t-values generated from the *robustlmm* R package and degrees of freedom (df = n-1). The *bacon* R package was implemented to obtain inflation-corrected p-values [53], and multiple test correction was performed using the Benjamini–Hochberg false discovery rate (FDR) method [54]. Biological effect size for DNA methylation (Δβ) per CpG was calculated as the difference in β value between the two timepoints, averaged across all individuals. CpGs with *bacon*-corrected FDR < 0.1 and Δβ >|0.03| were considered statistically significant.

### Differential maternal DNA methylation associated with gestational age at birth

To investigate DNA methylation patterns associated with gestational age at birth, we fitted separate robust linear models for the two pregnancy timepoints on β values of each of the 224,663 variable CpGs. Robust linear modeling was performed using the rlm() function in the *MASS* R package [55]. The regression model for the site-by-site EWAS is as follows:

$$\begin{aligned} &CpG\;\beta\;value\sim Gestational\;age\;at\;birth\;\left(in\;weeks\right)\\&+Maternal\;age+Cell\;type\;PCs\;1-2\end{aligned}$$

Biological effect size for DNA methylation (Δβ) was calculated by extracting the β coefficient of gestational age from the regression model and multiplying that with the range between the lower (5th percentile) and upper (95th percentile) quantiles of gestational age in our study, i.e., delta beta = β coefficient × (Gestational age at the upper quantile – Gestational age at the lower quantile). In doing so, we quantify the change in CpG methylation over a typical range (encompassing the middle 90% of the data) of gestational age. CpGs that passed the statistical cut-off of FDR adjusted p-values < 0.1 (equivalent to p-value 2.116e-06 at early pregnancy and 1.805e-05 at late-mid pregnancy) and Δβ >|0.03|, reflective of change in DNA methylation, were considered significantly associated with gestational age at birth. To explore if our significant findings were timepoint-specific or if they could potentially be identified across timepoints once the multiple testing burden is reduced, we tested the identified early pregnancy CpGs as candidates at the late-mid pregnancy timepoint, and the late-mid pregnancy CpGs at the early pregnancy, using the same model and thresholds as described above.

### Post-hoc characterization of identified early and late-mid pregnancy maternal DNA methylation associations with gestational age at birth

In efforts to characterize the identified DNA methylation associations, we first performed a contribution analysis [56] to assess the relevance of psychosocial and biological factors as it relates to maternal DNA methylation and gestational age. Second, we used the ARIES database and *ENmix* R package [57] to explore the influence of potential genetic variant effects on identified DNA methylation patterns (methylation quantitative trait loci (mQTLs)). Third, we grouped correlated, adjacent CpGs into biologically relevant units called co-methylated regions (CMRs) using the *CoMeBack* R package [58] and tested their association with gestational age at birth. Fourth, we carried out an overrepresentation analysis of chromatin states (promoters, enhancers, transcribed regions, repressed regions, and quiescent regions) among our exploratory EWAS findings using ChromHMM [59]. Fifth, we performed a lookup of our EWAS findings using EWAS atlas and EWAS catalogue to identify potential molecular mechanisms of epigenetic modifications associated with biological traits [60, 61]. Finally, we compared β values of these CpGs averaged across individuals in preterm versus term groups, to assess whether DNA methylation levels at the identified CpGs were altered in preterm pregnancies. Details of above mentioned post-hoc analyses are provided in Additional file 2.

### Secondary analyses of identified maternal blood DNA methylation findings in three external cohorts

To explore the uniqueness of our gestational age specific maternal blood DNA methylation associations, we leveraged DNA methylation data available in three external cohorts with socioeconomic and cultural settings different from our LMIC discovery cohort: i) Cebu Longitudinal Health and Nutrition Survey (CLHNS) [62], and two publicly available datasets ii) GSE114935 and iii) GSE224339 [10, 63]. The GSE114935 and GSE224339 cohorts comprise matched maternal blood DNA methylation quantified at two timepoints in pregnancy, i.e., the first/early second trimester and at delivery, while the CLHNS cohort includes DNA methylation profiled at a single timepoint, i.e., 22.86–29 weeks. We acknowledge that the timepoints measured in the GEO external cohorts do not perfectly correspond to the early and late-mid timepoints in the LMIC discovery cohort (Karachi), however, the 1 st trimester (11.6—18.9 weeks) timepoint in GSE224339 has some overlap with our tested timepoint, allowing us to explore our cell type and gestational age findings in different socioeconomic and cultural settings. Further, the two GEO datasets include DNA methylation measurements at delivery, which enabled us to explore our identified cellular and molecular changes beyond the late-mid pregnancy timepoint in our discovery. Information on cohort demographics is available in Additional file 1 – Supplementary Table 2.

For GSE114935 and GSE224339, raw IDAT files were downloaded from GEO. Sample quality, normalization, probe filtering and batch correction steps were performed as in discovery cohort. Sample matching at the two timepoints were confirmed using the SNP probes available on the 450 K and 850 K arrays. In the CLHNS cohort, sample quality checks were similarly performed, with additional steps such as visual inspection of outlier samples based on PCA. Similar probe filtering steps were carried out, followed by BMIQ normalization. For all three external cohorts, only women with newborns with gestational ages > 37 weeks was retained for subsequent analyses.

The extended IDOL reference was used to estimate proportions of twelve leukocyte subtypes; in the GEO datasets with two timepoints, cell type proportions at the two timepoints were further compared to detect any shifts cell type composition from first/early second trimester to delivery [48]. Additionally, in the two GEO datasets, maternal DNA methylation changes between the two timepoints were tested using a linear mixed-effect model (as described in Methods 2.7). In all three cohorts, DNA methylation patterns associated with gestational age at birth were tested independently at the corresponding timepoints using linear regression models (as described in Methods 2.8).

## Results

### Estimated neutrophil, natural killer cell, B memory, CD8 memory and CD4 memory cell proportions were significantly different between early and late-mid pregnancy timepoints

**Fig. 1:**
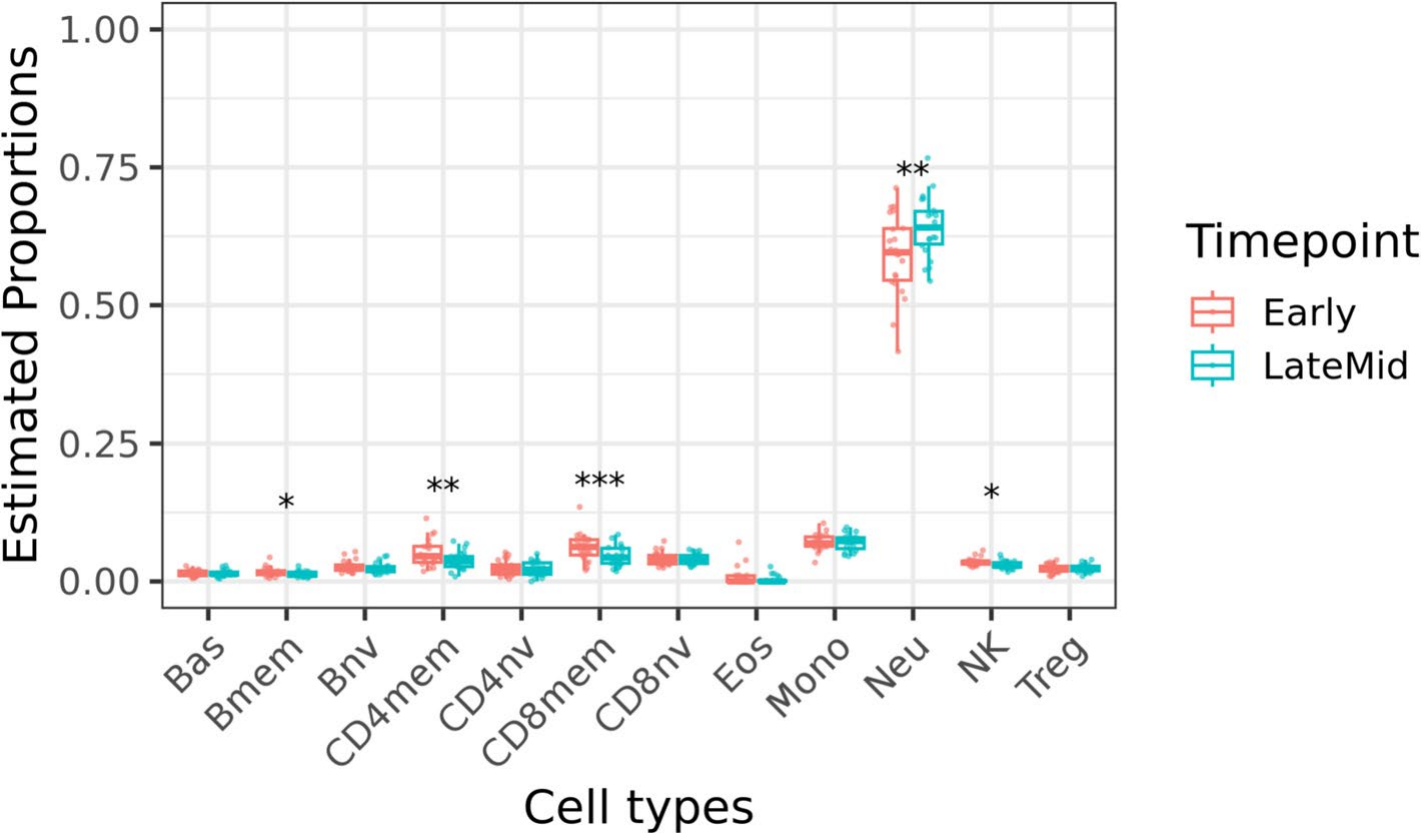
Differences in immune cell type proportions in maternal blood estimated at early and late-mid pregnancy timepoints. In the boxplots, the X-axis represents the twelve immune cell types investigated; the Y-axis represents cell type proportions inferred from the DNA methylation-based IDOL-2022 reference. * indicate the level of FDR-corrected p-value (* *p* ≤ 0.05, ** *p* ≤ 0.01, *** *p* ≤ 0.001)

To test changes in immune cell profiles across gestation, we used the proportions of 12 immune cell types estimated from DNA methylation at early and late-mid pregnancy timepoints using reference-based cell-type deconvolution*.* Overall, as expected, neutrophils were the most abundant cell type at both early and late-mid pregnancy timepoints. Pairwise t-tests identified significant differences in the estimated proportions of neutrophils (Bonferroni *p* = 0.0051, t = −3.1295, df = 21, Cohen’s d = −0.6672), natural killer (Bonferroni* p* = 0.0126, t = 2.7263, df = 21, Cohen’s d = 0.5812), B memory (Bonferroni* p* = 0.0192, t = 2.5355, df = 21, Cohen’s d = 0.5406), CD8 memory (Bonferroni* p* = 0.0003, t = 4.3159, df = 21, Cohen’s d = 0.9202) and CD4 memory (Bonferroni *p* = 0.0032, t = 3.3243, df = 21, Cohen’s d = 0.7087) cells between early and late-mid pregnancy (Fig. 1, Additional file 1- Supplementary Table 3). Of the cell types with statistically significant differences between timepoints, neutrophils had a marked increase at late-mid pregnancy timepoint, whereas NK, B memory, CD8 memory, and CD4 memory cells exhibited lower average proportions during late-mid pregnancy when compared to early pregnancy timepoint.

Further, leveraging the matched timepoints available in the two GEO external cohorts (Additional file 1—Supplementary Table 2), we assessed changes in immune cell proportions between first/early-second trimester and delivery, and compared these changes to those observed in our discovery (Additional file 1—Supplementary Table 4). Although the timepoints of the discovery and GEO external cohorts do not perfectly overlap, we still noted consistent immune cell type changes across the cohorts. Specifically, we noted that neutrophil proportions were markedly increased later in gestation, i.e., at delivery, whereas NK, B memory, CD8 memory, and CD4 memory cells exhibited lower average proportions at delivery when compared to the earlier first/early-second trimester timepoints. Besides these five immune cell types, we observed significant proportional changes in additional cell types from first/early-second trimester to delivery in the two GEO cohorts, an observation also noted in their corresponding studies [10, 63].

### DNA methylation levels in maternal blood predominantly remained unchanged between early and late-mid pregnancy timepoints

**Fig. 2:**
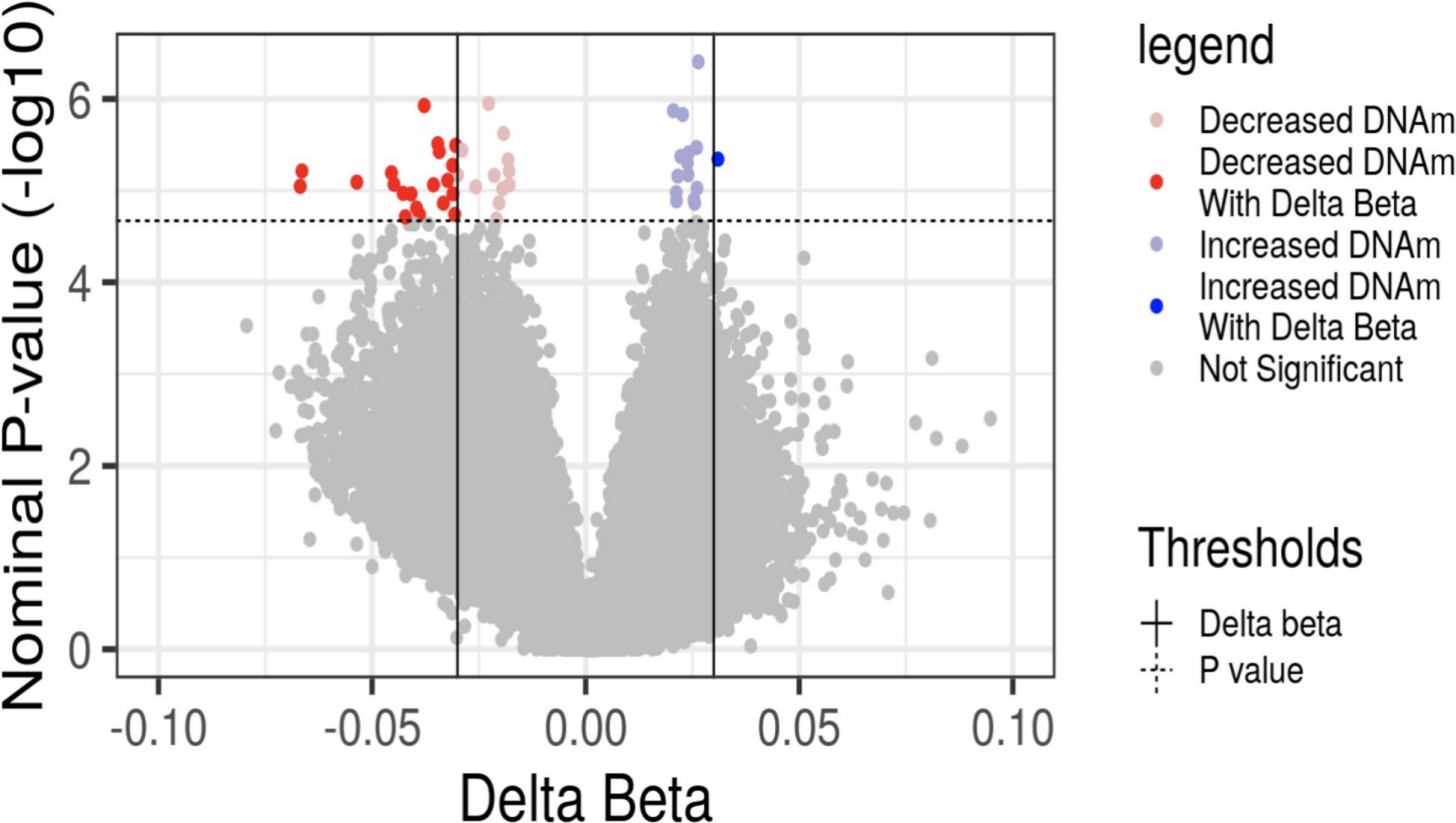
Differentially methylated CpGs between early and late-mid pregnancy timepoints in maternal blood, with individual as a random effect. Volcano plot of epigenome-wide differential DNA methylation analysis with timepoint of maternal blood collection, after adjustment for mother’s age and differences in estimated proportions of Bmem, CD4mem, CD8mem, neutrophils and NK cells between the two timepoints. The X-axis represents the difference in beta value per CpG between the two timepoints, averaged across all individuals (Δβ); the Y-axis represents the − log10(unadjusted bacon p-value). Dotted horizontal line indicates the threshold for bacon-corrected false discovery rate (FDR) adjusted significance, and vertical lines indicate the threshold for Δβ. The dark red and blue colors represent CpGs that meet both the bacon-corrected FDR < 0.1 and Δβ >|0.03| thresholds, while the lighter red and blue colors indicate CpGs that meet the bacon-corrected FDR < 0.1 criterion only

On comparing DNA methylation differences between the early and late-mid pregnancy timepoints, after adjusting for maternal age, cell types, and individual as a random effect to account for repeated measurements at the two timepoints, we identified a relatively small set of 21 CpGs (~ 0.01% of investigated array CpGs) at bacon-corrected FDR < 0.1 and Δβ > 3% that displayed DNA methylation change across the investigated timepoints, indicating that the maternal blood DNA methylation landscape remained largely unchanged when taking cell-type proportion changes into account. Of the CpGs that do change, with one exception (*cg07638723*), all CpGs (20 out of 21 CpGs), demonstrated a decrease in maternal blood DNA methylation levels over time, as these women progressed from early to late-mid pregnancy (Fig. 2, Additional file 1- Supplementary Table 5). Further, we noted that these small set of CpGs exhibited differences in DNA methylation levels between the tested timepoints in the GSE224339 and GSE114935 cohorts (Additional file 1—Supplementary Table 6).

### DNA methylation associations with gestational age at birth were pronounced in early pregnancy compared to late-mid pregnancy timepoint

**Fig. 3:**
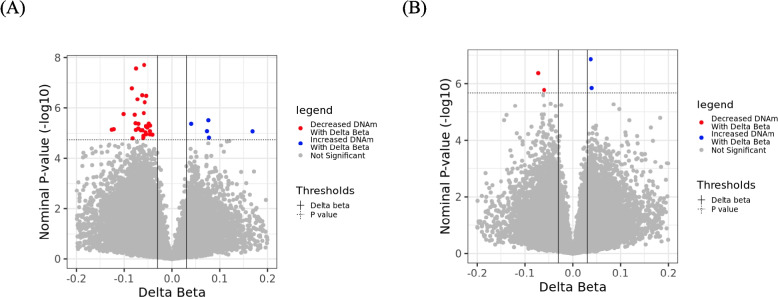
Differentially methylated CpGs in maternal blood associated with gestational age at birth in early and late-mid pregnancy. Volcano plot of epigenome-wide differential DNA methylation analysis with gestational age at birth measured at (**A**) early and (**B**) late-mid pregnancy, after adjustment for mother’s age and estimated cell-type principal components (PCs). The X-axis represents model coefficients of gestational age multiplied by the cohort range of gestational age between the 5th and 95th percentile (Δβ); the Y-axis represents the − log10(unadjusted *p*-value). Dotted horizontal line indicates the threshold for false discovery rate (FDR) adjusted significance, and vertical lines indicate the threshold for Δβ. The dark red and blue colors represent CpGs that meet both the FDR < 0.1 and Δβ >|0.03| thresholds

Using exploratory EWAS analyses to evaluate DNA methylation signatures in maternal blood associated with gestational age, we identified 37 CpGs in early and 4 CpGs in late-mid pregnancy that were predictive of gestational age at birth (FDR < 0.1, Δβ > 0.03) (Fig. 3 A and B), accounting for maternal age and cell type proportions. Of the 37 gestational age specific CpGs identified at the early pregnancy timepoint, 5 CpGs exhibited increased DNA methylation and 32 CpGs exhibited decreased DNA methylation with gestational age at birth. Similarly, at the late-mid pregnancy timepoint, of the 4 CpGs identified, 2 CpGs exhibited increased DNA methylation and 2 CpGs decreased in DNA methylation with gestational age at birth. (List of differentially methylated CpGs and annotated gene names are provided in Additional file 1-Supplementary tables 7 and 8). We found more gestational age associated CpGs at early pregnancy compared to late-mid pregnancy; however, these do not overlap with the associations identified at the late-mid pregnancy timepoint.

Further, we evaluated the specificity of the identified gestational age linked DNA methylation associations to early and late-mid pregnancy. Using the same models and statistical and biological thresholds (FDR < 0.1 and Δβ > 0.03), we tested each CpG discovered in the exploratory EWAS at the other pregnancy timepoint in which it was not detected. We noted that 31 out of 37 early pregnancy CpGs, and 3 out of 4 late-mid pregnancy CpGs, were not significant in the other timepoint, exhibiting distinct gestational age specific DNA methylation at the timepoints that they were identified in (Additional file 2-Supplementary Fig. 2, Additional file 1- Supplementary tables 9 and 10). To further assess this limited overlap, we applied a more relaxed threshold of nominal *p*-value < 0.01 to evaluate whether these CpGs were at least borderline significant in the other timepoint. Under this relaxed threshold, 32 out of 37 early pregnancy CpGs and all 4 late-mid pregnancy CpGs were still not significant at the other timepoint, suggesting that the associations observed were largely timepoint-specific and not simply the result of conservative statistical filtering.

### Gestational age specific DNA methylation associations in the LMIC discovery cohort were largely distinct from those in the three external cohorts

**Fig. 4:**
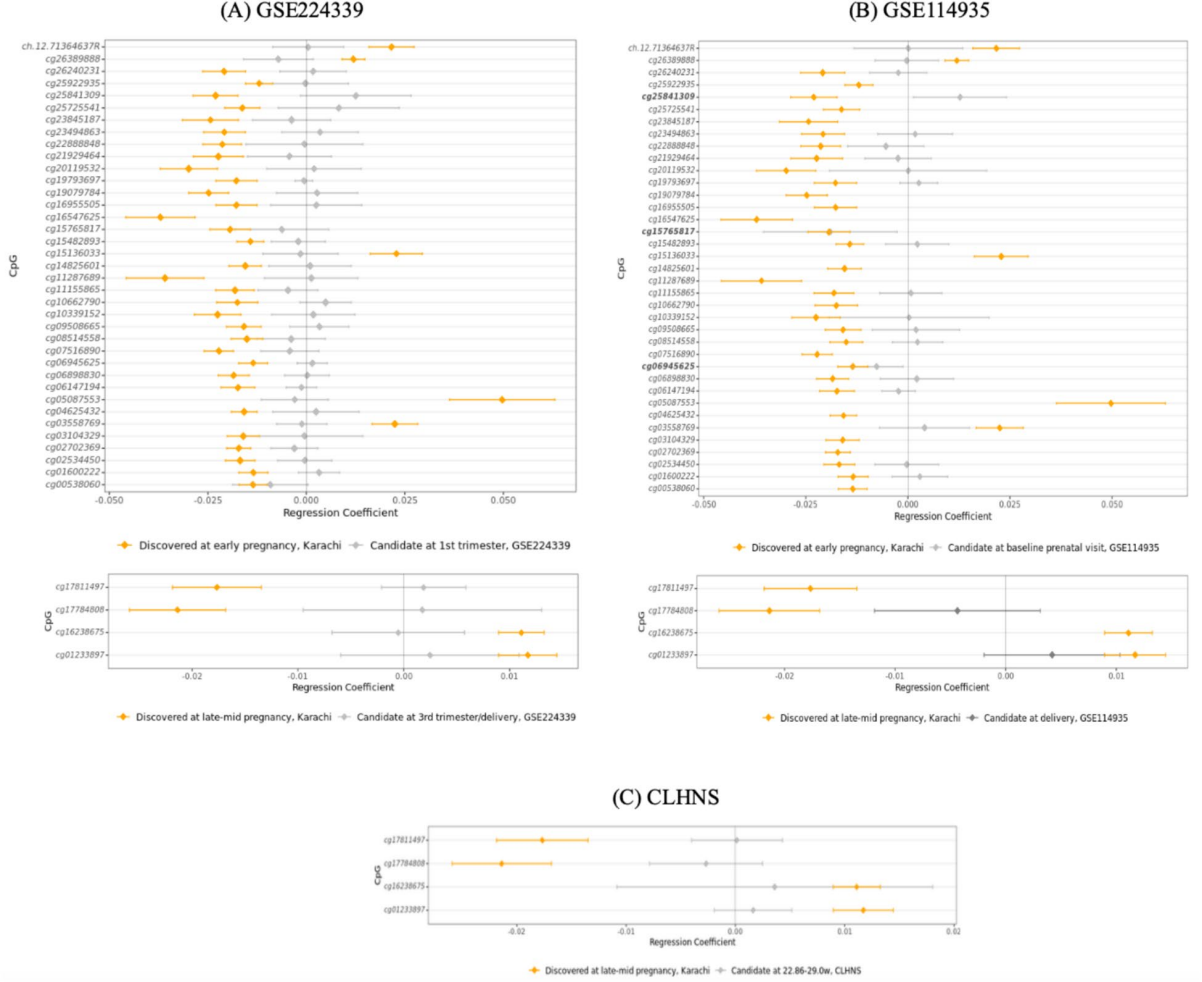
Gestational age specific CpGs identified in the discovery cohort were largely unique to the investigated LMIC setting compared to the three external cohorts. Forest plots display the regression coefficients of gestational age specific CpGs discovered at early and late-mid pregnancy in the Karachi cohort and tested as candidates at the corresponding timepoints in the (**A**) GSE224339, (**B**) GSE114935, and (**C**) CLHNS external cohorts. The X-axis represents the regression coefficient and 95% confidence intervals per CpG in the discovery (coloured in orange) and secondary (coloured in grey) analyses respectively; the Y-axis represents the gestational age specific CpGs that were identified in the discovery and subsequently tested at the corresponding timepoint in the external cohorts. The CpGs significantly associated with gestational age in both the discovery and external cohorts are highlighted in bold

We also assessed whether the gestational age specific DNA methylation signatures identified in our discovery cohort were unique to the LMIC setting compared to three external cohorts differing in socioeconomic and cultural settings. The gestational windows examined in the GEO external cohorts do not perfectly align with the tested timepoint in our LMIC discovery cohort, although there is some overlap. As expected, the gestational age specific CpGs identified in our LMIC discovery cohort largely showed no significant differential DNA methylation with gestational age in the three external cohorts even at a nominal p-value of 0.05 (Fig. 4, Additional file 1—Supplementary Table 11). Only three out of 21 tested CpGs (*cg06945625, cg15765817, cg25841309*) in GSE114935 were significantly associated with gestational age at baseline prenatal visit (< 12 weeks).

### Post-hoc characterization

#### SES score in early pregnancy and predicted IL-6 score in late-mid pregnancy contributed to the maternal DNA methylation associations with gestational age

Psychosocial, demographic, and biological variables, influential factors during pregnancy, were tested on the identified gestational age specific maternal DNA methylation associations using the contribution models described in Methods. We hypothesized that these important determinants of pregnancy, maternal prenatal mental health (pregnancy-related anxiety z-scores and depression z-scores at each timepoint), SES in early pregnancy, physiological burden (allostatic load) in early pregnancy, and DNA methylation-based IL-6 at each timepoint contribute to the association between maternal DNA methylation and gestational age. Predicted IL-6 score contributed 20.58% in DNA methylation of *cg01233897* (intergenic) at late-mid pregnancy, but there was no contribution noted at early pregnancy (Table 2). Additionally, during early pregnancy, SES contributed 34.58% in *cg16955505 (C14orf119* gene), 26.87% in *cg10662790* (*ADCY3* gene)*,* 23.83% in *cg08514558* (*PPIF* gene)*,* and 7.77% in *cg23845187* (intergenic)*,* with 23.26% median percent contribution, while there was no contribution noted at late-mid pregnancy. In contrast, maternal prenatal mental health and allostatic load were not significant contributors at either timepoints.

**Table 2:** SES score in early pregnancy and predicted IL-6 score in late-mid pregnancy contribute to maternal blood DNA methylation relation to gestational age at birth. (Only statistically significant findings with FDR < 0.1 are shown)

Variable	CpG	Gene	Variable *p*-value	Variable FDR	Variable coefficient (Base model)	Variable coefficient (Adjusted model)	Contribution (%)
SES score (early pregnancy)	*cg16955505*	*C14orf119*	0.0008	0.0104	−0.0177	−0.0116	34.58
	*cg10662790*	*ADCY3*	0.0007	0.0104	−0.0175	−0.0128	26.87
	*cg08514558*	*PPIF*	0.0001	0.0053	−0.0151	−0.0115	23.83
	*cg23845187*		0.0008	0.0936	−0.0177	−0.0116	7.77
Predicted IL-6 score (late-mid pregnancy)	*cg01233897*		0.0233	0.0930	0.0117	0.0093	20.58

#### Twelve of the 37 gestational age specific CpGs at early pregnancy were within co-methylated regions 

Proximally located CpGs often exhibit correlated DNA methylation patterns which might indicate a higher likelihood to function as a biologically relevant unit. We determined whether the gestational age specific CpGs identified from the EWAS analyses (early pregnancy: 37 CpGs, late-mid pregnancy: 4 CpGs) were located within contiguous, correlated CpG regions (or CMRs). Using the *CoMeBack* R package [58], we identified 70,715 CMRs ranging in size from 2 to 67 CpG probes at early pregnancy and 67,176 CMRs ranging in size from 2 to 59 CpG probes at late-mid pregnancy, with an overlap of 26,787 CMRs between the two timepoints.

**Fig. 5:**
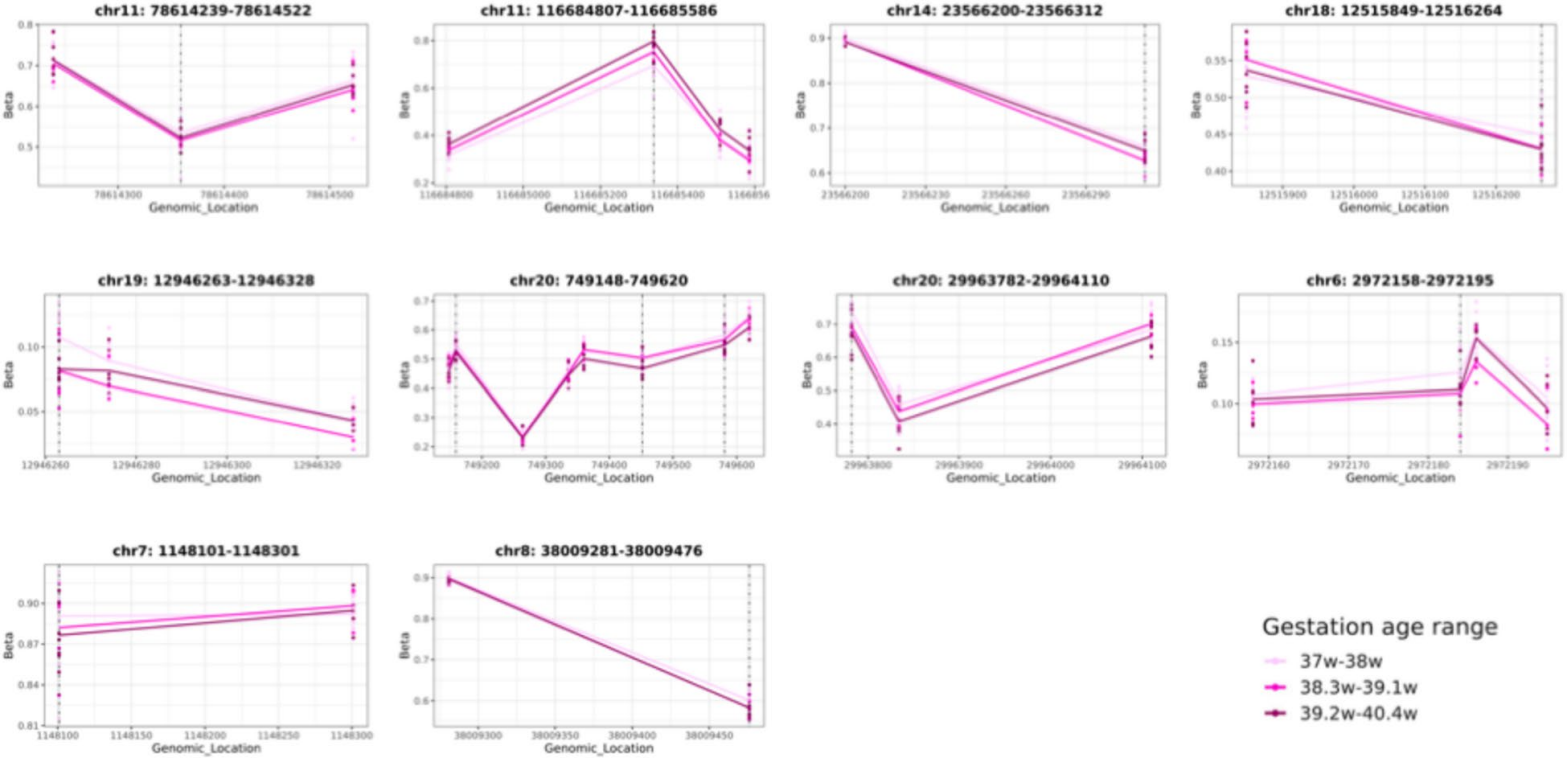
Of the 37 gestational age specific CpGs at early pregnancy, twelve mapped to co-methylated regions (CMRs) and these CMRs were also associated with gestational age at birth at FDR < 0.1 and Δβ >|0.03|. Line plots depict the beta distribution of all CpGs within a CMR, which is significantly associated with gestational age at early pregnancy*.* The gestational age at birth variable is divided into tertiles for plotting (37-38 weeks, 38.3–39.1 weeks, 39.2–40.4 weeks). The dashed vertical lines indicate the respective genomic positions of the early pregnancy EWAS findings within the CMR

At early pregnancy timepoint, of the identified 37 gestation-age associated CpGs, 12 CpGs mapped to 10 unique CMRs (median CMR length: 3 CpGs), while 25 CpGs did not map to any CMR (Additional file 1- Supplementary Table 12). On the other hand, none of the four identified CpGs at late-mid pregnancy timepoint mapped to a CMR. Comparing the 10 CMRs identified at early pregnancy to the 26,787 CMRs that overlapped between the two timepoints, we noted that three CMRs (i) *cg14276388, cg16955505*, ii) *cg19793697, cg02605370, cg20402675*; and iii) *cg11287689, cg01915090, cg09037093*) were uniquely identified only in early pregnancy, while the remaining seven were identified in both pregnancy timepoints. The subset of 10 CMRs at early pregnancy were further tested as regions for their association with gestational age at birth using the same model as described in Methods*.* Seven of the 10 early pregnancy CMRs had significant associations with gestational age at FDR < 0.1 & Δβ >|0.03| (Fig. 5).

#### Limited genetic influence and no significant enrichment of chromatin states on the identified maternal DNA methylation associations with gestational age

Certain genetic variants regulate DNA methylation patterns at specific CpGs (mQTLs) [64]. In the absence of directly measured SNP information, potential mQTLs can be examined by inspecting the distribution of DNA methylation levels at CpGs, with a bimodal or a trimodal distribution, typically suggesting the influence of genotype (i.e., AA, Ab, bb) on DNA methylation patterns. Using the nmode() function in the *ENmix* R package, we did not identify any potential mQTLs at the early or late-mid pregnancy timepoints. However, taking advantage of the existing ARIES mQTL database, we identified 6 of the 37 early pregnancy CpGs and 2 of the 4 late-mid pregnancy CpGs as potential pregnancy-specific mQTLs, although it should be noted that individuals from South Asian ancestry are underrepresented in this database (Additional file 2-Supplementary Fig. 3). As chromatin states play a role in gene regulation by DNA methylation, we used ChromHMM maps and identified neutrophil- and mononuclear cell-specific chromatin states underlying our CpG associations. Overrepresentation analysis, based on derived mononuclear cell- and neutrophil- specific ChromHMM maps, identified no significantly enriched chromatin states at FDR < 0.1 at early and late-mid pregnancy timepoints (Additional file 2-Supplementary Fig. 4).

#### Average cg06147194 methylation was significantly different between term and preterm at early pregnancy

We next explored whether DNA methylation patterns in maternal blood collected from mothers delivering term were also altered in those with preterm deliveries. For this, we leveraged data available for four preterm samples from the Sindhi cohort that were excluded in the previous EWAS and compared average β values of identified gestational age associated CpGs between term and preterm samples. Of the 37 CpGs associated with gestational age at early pregnancy, one CpG (*cg06147194,* annotated to gene *FDXR*) exhibited a significant difference in average DNA methylation between mothers delivering preterm and term (FDR = 0.026, Δβ = 0.0774). None of 4 CpGs associated with gestational age at late-mid pregnancy displayed DNA methylation differences between preterm and term (Additional file 2-Supplementary Fig. 5).

## Discussion

Perinatal research is largely confined to populations in high-income countries, with primarily individuals of European ancestry. Obstetric, psychological, socioeconomic, and cultural conditions surrounding pregnancy and childbirth in high-income populations differ significantly from those in LMIC. Owing to these differences, pregnant women in LMIC exhibit an increased prevalence of mental health conditions, socioeconomic distress, and perceived stress, all of which can negatively impact the trajectory of pregnancy and its outcomes [65]. Pregnant women in LMIC also have limited access to healthcare services which increase their likelihood of experiencing an adverse pregnancy outcome. To address this important gap, we longitudinally sampled pregnant women from an LMIC setting, across four hospital sites in Karachi, Sindh, Pakistan, and leveraged maternal blood collected twice during their term pregnancy (early: 10–19 weeks and late-mid: 22–29 weeks). We noted significant immune cell proportion differences between the investigated timepoints, however, the DNA methylome remained largely unchanged when accounting for these immune cell changes. We next explored if the maternal DNA methylome, a proxy for the in utero developmental environment of the fetus, was predictive of gestational age. We noted timepoint-specific DNA methylation signatures associated with gestational age, with a more pronounced molecular signature at the early timepoint.

Proportional changes in immune cell types over the course of pregnancy have been previously established [6, 7], predominantly in populations sampled from high income countries, and cell type composition in heterogenous samples such as whole blood do explain a large fraction of the variation in DNA methylome [8–10]. Therefore, we first evaluated immune cell type profiles at the two timepoints and found neutrophils to be the most abundant cell type at both early and late-mid pregnancy timepoints. This is consistent with previous findings of increased white blood cell counts during pregnancy, with studies describing mild neutrophilia [66, 67]. On comparing the two timepoints, we noted average estimated neutrophil proportions were higher in the late-mid pregnancy timepoint, likely explained by the developing maternal-placental circulatory interface in the second trimester. Of the other investigated cell types, NK, B memory, CD8 memory, and CD4 memory cells were also significantly different between the two timepoints. Unlike neutrophils, these cell types exhibited reduced proportions in the late-mid pregnancy timepoint. Similar changes in immune cell populations were also noted in the external cohorts which varied in sociodemographic characteristics compared to our investigated LMIC cohort, suggesting that these normative immune changes may be indicative of a shift to a maternal immune quiescent state to mediate fetal tolerance and promote pregnancy success [68].

When examining longitudinal DNA methylation patterns coarsely across pregnancy, we observed changes in individuals between the early and late-mid pregnancy time points when accounting for cell type proportion changes. While these signatures were only noted in a small percentage (0.01%) of the investigated CpGs, we observed a consistent change in direction in the same set of CpGs when assessed in the external GEO longitudinal cohorts. Limited changes to the DNA methylome during pregnancy was also reported in a previous longitudinal study, where DNA methylation changes in maternal blood were examined at puberty, conception, and gestation, and no significant changes were noted during gestational transition from early to late pregnancy [69]. It is therefore plausible that DNA methylation changes in this small set of CpGs are important for biological processes characteristic of a successful pregnancy, however other epigenetic mechanisms may be integral as well and future research should explore this. It should also be noted that our speculation is restricted to the CpGs investigated on the Illumina 850 K array, which covers only ~ 3% of the genome.

Our exploratory EWAS analyses revealed specific maternal blood DNA methylation signatures predictive of gestational age at the two investigated timepoints, with limited influence of genetic variants on the identified DNA methylation signatures. We noted a more pronounced DNA methylation signature at early pregnancy, with most identified associations (31 out of 37 early pregnancy and 3 out of 4 late-mid pregnancy CpGs) specific to the timepoints in which they were discovered in, likely reflective of time point-specific maternal programming to support fetal development. A small number of gestational age specific CpGs (6 out of 37 early pregnancy and 1 out of 4 late-mid pregnancy CpGs) were identified at the other pregnancy timepoint in which they were not previously detected, but these associations were of larger effect size in the discovery timepoint. This limited overlap across timepoints may represent maternal adjustments specific to the gestational stage, though it could also be partly attributed to insufficient statistical power to detect shared associations. Overall, our findings captured a pronounced gestational age specific DNA methylation signature at the early 10–19 weeks in pregnancy, indicating that this window of time may be more appropriate in testing DNA methylation associations to gestational age at birth. Specifically, our early pregnancy findings in maternal blood were annotated to solute carrier superfamily genes (*SLC52A3, SLC37A1, SLC12A5 and SLC52A3*), which are expressed in the placenta to facilitate nutrient uptake and transfer and regulate fetal development [70]. We also noted that a proportion of the identified CpGs were related to genes involved in immunity (*TMPRSS2, IGSF21, SPIRE1,* and *SERPINB6*) and neurogenesis (*NUDC, ODZ4, DAB1, SNCA,* and *A2BP1*), emphasizing their potential biological relevance during the early 10–19 weeks in gestation – possibly both in supporting infant development during pregnancy and preparing for motherhood after birth. As we compared our maternal blood findings to existing gestational age specific EWAS literature in fetal and birth tissues [15–21], we noted a minimal concordance of 5 CpGs (*cg03104329, cg23494863, cg25725541, cg15482893,* and *cg23494863*). Of these, CpGs annotated to genes *SERPINB6, STAR* and *SPIRE* also mapped to CMRs at the early pregnancy timepoint indicating their potential to act together as a functional unit.

We also compared our gestational age specific associations to three external cohorts, which measured maternal DNA methylation at approximately the same timepoints compared to those in the discovery cohort. As anticipated, our findings largely did not show differential DNA methylation with gestational age at birth in the external cohorts, suggesting that they were largely unique to our LMIC discovery setting. Owing to the specific challenges and conditions faced by pregnant women in Karachi, Sindh, Pakistan compared to those in other investigated settings, it is not surprising that these changes in the maternal epigenome were not observed beyond the discovery LMIC setting. Further, alterations in genetics, lifestyle and environmental conditions, and technical factors such as processing facility and sample handling may have contributed to the observed differences.

The biological basis underlining the association between maternal DNA methylation and gestational age is not well characterized. While the role of psychosocial and biological factors in pregnancy is established [27, 29–35], the influence of these factors on gestational age specific differential DNA methylation is understudied. Evaluating these factors in the context of the maternal epigenome is important as they may be reflective of both the mother’s cumulative life experience and her existing physiological state [71]. At the early pregnancy timepoint, SES, an indicator of prenatal stress burden due to potential financial hardship, contributed to gestational age specific DNA methylation associations at four CpGs: *cg16955505* (C14orf119 gene), *cg10662790* (*ADYC3* gene), *cg08514558* (*PPIF* gene), and *cg23845187* (not annotated to a gene), however, this effect was not noted at the late-mid pregnancy timepoint. As maternal investment increases with progressing gestational age, there is a dampening of stress response that occurs to protect the growing fetus [24], which may explain why we do not note any SES effects on DNA methylation associations at the later timepoint. Contrastingly, predicted IL-6 score contributed to the DNA methylation association at *cg01233897* (intergenic) at the late-mid pregnancy timepoint, and this effect was not noted at the early pregnancy timepoint. Our ability to detect IL-6 effects at the late-mid pregnancy is attributable to its higher estimated levels at that timepoint when compared to early pregnancy, as also seen in previous investigations [72]. IL-6 begins to cross the placental barrier, separating the maternal and fetal compartments, starting mid-gestation [73–75], suggesting that mid-gestation may be an appropriate timepoint to explore the contribution of inflammatory markers such as IL-6 to gestational age specific DNA methylation levels.

While our study resolves important gaps in current perinatal research in LMIC settings, there are a few limitations. First, our EWAS analyses may be limited by the small sample size of 22 pregnant women, all of whom carried their pregnancies to term. To address this limitation, we applied a variability filter to identify informative CpGs, which, while potentially excluding borderline-variable probes due to the limited sample size, helped reduce noise and enhance statistical power. Additionally, we used a modest FDR threshold of 0.1 and only selected biologically relevant CpGs having effect size greater than 3%, as it has been previously shown that using an effect size cutoff along with an FDR threshold is more likely to capture biologically meaningful signatures and improve replication potential [76]. Second, we confirmed that our DNA methylation findings were reflective of term pregnancies by comparing these associations to preterm cases. We found one CpG (*cg06147194*) linked to the mitochondrial flavoprotein gene *FDXR* (ferredoxin reductase) with differential DNA methylation in preterm pregnancies. While an *FDXR* genetic variant in the fetal genome has been linked to a common preterm birth condition, preterm premature rupture of the membranes (PPROM) [77], only 4 preterm samples were available for this exploratory analysis, therefore we acquiesce that we are not powered to make any definitive statements about how our observations hold up in preterm births. Third, we relied upon a DNA methylation-based algorithm to estimate cell type proportions and IL-6 levels, as direct measurements from maternal blood were not available. While good concordance between cell counts and bioinformatically predicted cell type proportions has been reported [78–80], these tools were not trained on samples from LMIC settings. Fourth, we cannot rule out that some of the identified DNA methylation signatures may be influenced by genetic variants, however in the absence of genotyping data we relied on *in-silico* mQTLs tools available in the R package *ENmix* and the ARIES pregnancy mQTL database. Fifth, we acknowledge that the composite SES score used in the study may be confounded with ethnicity, social experiences, and other social determinants of health. However, all participants were recruited from the same city Karachi and are representative of the same South Asian (SAS) super-population, therefore it is less likely that our EWAS findings are driven by ethnic differences. Finally, the educational attainment of our cohort was slightly higher than the national average, likely reflecting the sociodemographic characteristics of the geographic region from which participants were recruited. While this may not fully capture the broader socioeconomic continuum of the national population, the cohort nonetheless represents a relevant and often underrepresented segment of the population, providing a valuable foundation for future research in more demographically diverse populations across LMIC settings.

The current study's findings provide insight into time-specific molecular changes in maternal blood predictive of gestational age in women from Karachi, Sindh, Pakistan, acknowledging the complex socioeconomic and healthcare landscapes that classify Pakistan as a LMIC. We employ the LMIC classification in our research not only to describe economic status, but as a critical framework to analyze the interaction of social determinants of health and epigenetic modifications across pregnancy [69]. However, we urge future research to leverage the LMIC classification to highlight the specific challenges and conditions faced by pregnant women across political, cultural, and socioeconomic spectrums. This will enable not only a more refined understanding of the timing of epigenetic influences in pregnancy on gestational age, but also how these changes are intricately tied to the broader socioeconomic realities of living in a LMIC setting and illuminate both the shared and unique epigenetic patterns that arise in specific LMIC contexts. Additionally, future studies investigating whether DNA methylation signatures observed in healthy term pregnancies, such as ours, are altered in adverse conditions like preterm birth will facilitate understanding of epigenetic markers with clinical relevance to pregnancy outcomes.

## Conclusion

Conducted in a challenging LMIC setting, the present study highlights changes in immune cell and DNA methylation profiles in maternal blood over the course of gestation, although the vast majority of the measured methylome remains unchanged. Additionally, identified DNA methylation associations with gestational age are timepoint-specific, with a more pronounced signature at early pregnancy. These overall changes in maternal blood can create a framework of baseline changes characteristic of uncomplicated healthy pregnancies and can be further used to assess fluctuations in pregnancy. Considering the complex biological milieu of maternal blood as an environment which shapes and supports biological processes for the growing fetus, understanding DNA methylation changes in this environment in relation to gestational age at specific periods during pregnancy can inform us of the molecular basis of variation in normal fetal development.

## Supplementary Information

Supplementary Material 1.

Supplementary Material 2.

## Data Availability

The datasets analyzed during the current study are available in the Gene Expression Omnibus repository, GEO accessions: GSE290152, GSE114935, and GSE224339.
