# Supplementary material for: Distinct maternal DNA methylation associations with gestational age at early and late-mid term pregnancy in a low- and middle-income country: evaluation of biological, genetic, and psychosocial contributors
Source: BMC Pregnancy Childbirth. 2025 Oct 10;25:1062. doi: 10.1186/s12884-025-08037-6 (PMC12512460; doi:10.1186/s12884-025-08037-6)

**Distinct maternal DNA methylation associations with gestational age at early and late-mid term pregnancy in a low- and middle-income country: Evaluation of biological, genetic, and psychosocial contributors**

Marcia Smiti Jude * ^1,2^, Chaini Konwar * ^1,2^, Robyn J McQuaid ^3,4^, Farooq Ghani ^5^, Nazneen Islam ^6^, Sharifa Lalani ^7^, Sarah M. Merrill ^1,8^, Fizza Fatima ^1,2^, Julia L. MacIsaac ^1,2^, Ntonghanwah Forcheh ^9^, Calen P. Ryan ^10^, Nanette R. Lee ^11^, Christopher W. Kuzawa ^12^, Michael S. Kobor ^2,13^, Shahirose Sadrudin Premji ^14^, Maternal-infant Global Health Team (MiGHT) – Collaborators in Research †

* These authors contributed equally and should be recognized as co-first authors. Please address all correspondence concerning this article to these authors at [marcia.jude@bcchr.ca](mailto:marcia.jude@bcchr.ca) or [ckonwar@bcchr.ca](mailto:ckonwar@bcchr.ca).

^1^ BC Children’s Hospital Research Institute (BCCHR), 950 West 28th Avenue, Vancouver, BC, V5Z 4H4, Canada

^2^ The Department of Medical Genetics, Faculty of Medicine, University of British Columbia, Vancouver, BC, V6T 1Z3, Canada

^3^ Department of Neuroscience, Carleton University, Ottawa, Ontario, Canada

^4^ University of Ottawa Institute of Mental Health Research, Ottawa, Ontario, Canada

^5^ Department of Pathology and Microbiology, Aga Khan University, Karachi, Sindh, Pakistan

^6^ Molecular Pathology, Clinical Laboratory Medicine, Aga Khan University Hospital, Karachi, Sindh, Pakistan

^7^ School of Nursing and Midwifery, Aga Khan University, Karachi, Sindh, Pakistan

^8^ Department of Psychiatry and Human Behavior, The Warren Alpert Medical School at Brown University, Providence, Rhode Island, USA

^9^ School of Nursing, Faculty of Health Sciences, Queen’s University, Kingston, Ontario, Canada

^10^ Robert N. Butler Columbia Aging Center, Mailman School of Public Health, Columbia University, New York, NY 10032, USA

^11^ Office of Population Studies, University of San Carlos, Talamban, Cebu City 6000, Philippines

^12^ Department of Human Evolutionary Biology, Harvard University, Cambridge, MA 02138, USA

^13^ The Edwin S.H. Leong UBC Healthy Aging Chair—A UBC President’s Excellence Chair, University of British Columbia, Vancouver, British Columbia, Canada

^14^ Sally Smith Chair in Nursing, School of Nursing, Faculty of Health Sciences, Queen’s University, Kingston, Ontario, Canada

† **Maternal-infant Global Health Team (MiGHT) – Collaborators in Research (*Affiliations at the time of the study)**

Shahirose Sadrudin Premji ^14^, Neelofur Babar ^15^, Aliyah Dosani ^16^, Ntonghanwah Forcheh ^9^, Farooq Ghani ^5^, Imtiaz Jehan***** ^17^, Sharifa Lalani ^7^, Nicole Letourneau ^18^, Mohamoud Merali ^19^, Ayesha Mian* ^20^, Joseph Wangira Musana ^21^, Christopher T. Naugler * ^22^, Sidrah Nausheen ^15^, Christine Okoko* ^23^, Geoffrey Omuse ^24^, Saima Sachwani ^7^, Pauline Samia ^25^, Kiran Shaikh* ^7^, Rozina Shazad* ^26^, Salima Sulaiman ^27^, Sikolia Wanyonyi ^21^, Ilona S. Yim ^28^

^15^ Department of Obstetrics and Gynecology, Aga Khan University, Karachi, Sindh, Pakistan

^16^ School of Nursing and Midwifery, Mount Royal University, Calgary, Alberta, Canada

^17^ Department of Community Health Sciences, Aga Khan University, Karachi, Sindh, Pakistan

^18^ Faculty of Nursing, University of Calgary, Calgary, Alberta, Canada

^19^ Department of Counselling & Clinical Psychology, Aga Khan University Hospital, Nairobi, Kenya

^20^ Department of Psychiatry, Aga Khan University, Karachi, Sindh, Pakistan

^21^ Department of Obstetrics and Gynecology, Aga Khan University Hospital, Nairobi, Kenya

^22^ Pathology and Laboratory Medicine, Faculty of Medicine, University of Calgary, Calgary, Alberta, Canada

^23^ Aga Khan University Hospital, Nairobi, Kenya

^24^ Department of Pathology, Aga Khan University Hospital, Nairobi, Kenya

^25^ Department of Pediatrics and Child Health, Aga Khan University, Nairobi, Kenya

^26^ Quality and Patient Safety Department of Outreach, Aga Khan University Hospital, Karachi, Sindh, Pakistan

^27^ Faculty of Applied Health Sciences, Brock University, St. Catherines, Ontario, Canada

^28^ Department of Psychological Science, University of California, Irvine, Irvine, CA, USA

**SUPPLEMENTARY METHODS**

**1. Cohort recruitment and selection criteria**

The pilot cohort of 40 pregnant women in the current study is a representative sub-sample drawn from a larger population-based cohort comprising 1861 women recruited from Sindh, Pakistan. Women were sampled across four secondary care sites of the Aga Khan Hospital for Women and Children (AKHWC) – Garden, Karimabad, Kharadar, and Hyderabad. Pregnant women with a singleton pregnancy at 10-19 weeks gestation (estimated based on last menstrual period) were included in this study if they also were willing to return for a second assessment at 22-29 weeks gestation (minimum 10 week between visits), planned to deliver at AKHWC, and spoke one of Urdu, Sindhi, or English. Women who conceived through artificial reproductive technologies, used psychotropics, and reported pre-pregnancy medical conditions, including but not limited to, diabetes mellitus, thyroid disorder, mental health disorders, and HIV/AIDS were excluded from this study. None of the women self-reported cigarette smoking 12 months prior to or during pregnancy.

**2.1. Maternal prenatal distress measures**

Pregnancy-related anxiety was assessed using the pregnancy-related anxiety 10-item, 4-point Likert scale (range 10-40; cut-off 22; Cronbach’s α=.78) which evaluates feelings related to health during pregnancy, health of fetus/infant, and labor and delivery.

Depression was measured using the Edinburgh Perinatal Depression Scale (EPDS), which comprises 10-items assessed on a 4-point Likert (range 0-30; cut-off 10; across 7 studies spanning 15 countries (some LMIC) Cronbach’s α = .73-.87; 3-12 weeks test-retest = .53-.74; split-half = .73-.83).

**2.2. Socioeconomic status (SES) composite score estimation**

Information on individual level SES indicators including parents’ education, parents’ occupation, and household income were collected through questionnaires. These measures were described as categories on an ordinal scale from which participants chose the appropriate category. Parents’ educational levels were categorized as follows: 0 = No formal education, 1 = Less than primary school, 2 = Primary school completed, 3 = Secondary/High school completed, 4 = College/University completed, and 5 = Postgraduate degree. Parents’ employment status was categorized as follows: 0 = Student or Unemployed, 1 = Homemaker or Retired, and 2 = Employed. Household income levels were categorized as follows: 0 = Less than Rs 5,000, 1 = Rs 5,000–10,000, 2 = Rs 10,001–20,000, 3 = Rs 20,001–40,000, 4 = More than Rs 40,000, and "Missing" for responses of "Don't know / Prefer not to say". Subsequently the ranks assigned to these categories were summed to form the composite SES score that essentially captures the inherent associations between the different SES indices.

**3. Post-hoc analyses**

**3.1. Contribution of psychosocial and biological stress load in the association between maternal DNA methylation and gestational age at birth**

Pregnancy-related maternal distress, allostatic load, SES indices, and maternal inflammation, as reported in previous literature, contribute to the success of pregnancy ^1–7^. These factors were individually tested for their contribution to the association between maternal DNA methylation and gestational age at the early and late-mid pregnancy timepoints, using a contribution analysis ^8^. For every CpG discovered in the EWAS analyses, the percent contribution of the hypothesized contributing variable was calculated by comparing the base model with the adjusted model as shown below:

1. the base model (same as the linear regression used for the EWAS above):

CpG β value ~ Gestational age (in weeks) + Mother’s age + Cell-typePC1-2

1. the adjusted model, including the contributing variable being tested:

CpG β value ~ Contributing Variable + Gestational age (in weeks) + Mother’s age + Cell-typePC1-2

1. the percent contribution of each tested variable, determined using the β coefficients of gestational age from the base and adjusted models above:

Percent Contribution for each significant CpG = [(Coeff_base_ model – Coeff_adj_ model)/ Coeff_base_ model]*100

For both (i) and (ii), multiple test correction was performed using the Benjamini–Hochberg false discovery rate (FDR) method ^9^. A similar statistical cut-off of FDR<0.1 was applied.

**3.2. Identification of potential genetic variant effects in gestational age associated maternal DNA methylation signatures**

To identify the influence of potential genetic variant effects on DNA methylation, the nmode() function from the ENmix R package was implemented, with the default distance between modes greater than 0.2 in methylation beta value. This function identifies CpGs whose DNA methylation values exhibit a multimodal distribution, particularly a tri-modal pattern, suggesting DNA methylation values at those CpGs are likely influenced by nearby single nucleotide polymorphisms (SNPs), termed as methylation quantitative trait loci (mQTL). Additionally, the ARIES mQTL database (http://www.mqtldb.org) was used to identify pregnancy-related mQTLs that have been previously reported in literature.

**3.3. Identification of co-methylated regions (CMRs) in maternal blood associated with gestational age at birth**

Adjacent CpGs located within 400bp of one another typically have correlated methylation states and can be grouped together as a single unit called co-methylated region (CMR) that may be interpreted in terms of biological function. CMRs were constructed using the Co-methylation with genomic CpG background (CoMeBack) method ^10^. Here, a CMR was called when the genomic distance between two adjacent array probes was no more than 1 kb (with unmeasured intermittent genomic CpGs having 400 bp-density) and all pairs of adjacent probes had >0.40 Pearson correlation across individuals. Array probes that did not meet these criteria were considered ‘singleton’. For each identified CMR, a composite methylation measure per individual was also calculated as the probe median methylation level.

Subsequently, CpGs discovered in the EWAS analyses were analyzed to see if they mapped to identified CMRs, and if so, the association between CMR composite β measure and gestational age at birth was evaluated using the same model:

CMR composite β ~ Gestational age (in weeks) + Mother’s age + Cell-typePC1-2

**3.4. Chromatin-state annotations of identified gestational age specific maternal DNA methylation associations**

Over representation of chromatin states (promoters, enhancers, transcribed regions, repressed regions, and quiescent regions) among EWAS findings was carried out using ChromHMM ^11^. The 18-state ChromHMM annotation obtained from the Roadmap Epigenomics database was overlapped with 850K array annotation to account for background CpGs on the array. Gene names for the EWAS-identified CpGs were obtained, and enriched chromatin states were identified at FDR-corrected p-value <0.1.

**3.5. Analyzing gestational age associated CpGs identified in term pregnancy, in maternal blood of those delivering preterm**

To assess whether DNA methylation levels at the identified CpGs were altered in preterm pregnancies, we compared β values of these CpGs averaged across individuals in preterm versus term groups. Non-parametric Wilcoxon test, using the wilcox_test() function in the rstatix R package, was carried out to compare the term (n=22) and preterm (n=4, gestational age < 37 weeks) groups at early and late-mid pregnancy. DNA methylation differences (Δβ) between the two groups were then calculated by subtracting the average β value of preterm from the average β value of term on an individual CpG basis.

**REFERENCES**

1. PrabhuDas, M. *et al.* Immune mechanisms at the maternal-fetal interface: perspectives and challenges. *Nat. Immunol.* **16**, 328–334 (2015).

2. Wadhwa, P. D., Entringer, S., Buss, C. & Lu, M. C. The Contribution of Maternal Stress to Preterm Birth: Issues and Considerations. *Clin. Perinatol.* **38**, 351–384 (2011).

3. Glynn, L. M., Schetter, C. D., Hobel, C. J. & Sandman, C. A. Pattern of perceived stress and anxiety in pregnancy predicts preterm birth. *Health Psychol. Off. J. Div. Health Psychol. Am. Psychol. Assoc.* **27**, 43–51 (2008).

4. Doktorchik, C. *et al.* Patterns of change in anxiety and depression during pregnancy predict preterm birth. *J. Affect. Disord.* **227**, 71–78 (2018).

5. Kim, M. K. *et al.* Socioeconomic status can affect pregnancy outcomes and complications, even with a universal healthcare system. *Int. J. Equity Health* **17**, 2 (2018).

6. Sow, M., Raynault, M.-F. & De Spiegelaere, M. Associations between socioeconomic status and pregnancy outcomes: a greater magnitude of inequalities in perinatal health in Montreal than in Brussels. *BMC Public Health* **22**, 829 (2022).

7. Premji, S. S. *et al.* Prenatal allostatic load and preterm birth: A systematic review. *Front. Psychol.* **13**, 1004073 (2022).

8. Mayne, J. Contribution analysis: Coming of age? *Evaluation* **18**, 270–280 (2012).

9. Benjamini, Y. & Hochberg, Y. Controlling the False Discovery Rate: A Practical and Powerful Approach to Multiple Testing. *J. R. Stat. Soc. Ser. B Methodol.* **57**, 289–300 (1995).

10. Gatev, E., Gladish, N., Mostafavi, S. & Kobor, M. S. CoMeBack: DNA methylation array data analysis for co-methylated regions. *Bioinformatics* **36**, 2675–2683 (2020).

11. Ernst, J. & Kellis, M. Chromatin-state discovery and genome annotation with ChromHMM. *Nat. Protoc.* **12**, 2478–2492 (2017).

**SUPPLEMENTARY FIGURES**

**Supplementary figure 1**: DNA methylation based IL-6 scores estimated at the early and late-mid pregnancy timepoints show moderate correlation. (A) Box plot showing difference in average IL-6 scores predicted at early and late-mid pregnancy timepoints. pvalue was calculated using a paired t-test. (B) Scatter plot depicting the Pearson correlation between predicted IL-6 scores at early and late-mid pregnancy timepoints.

(A)


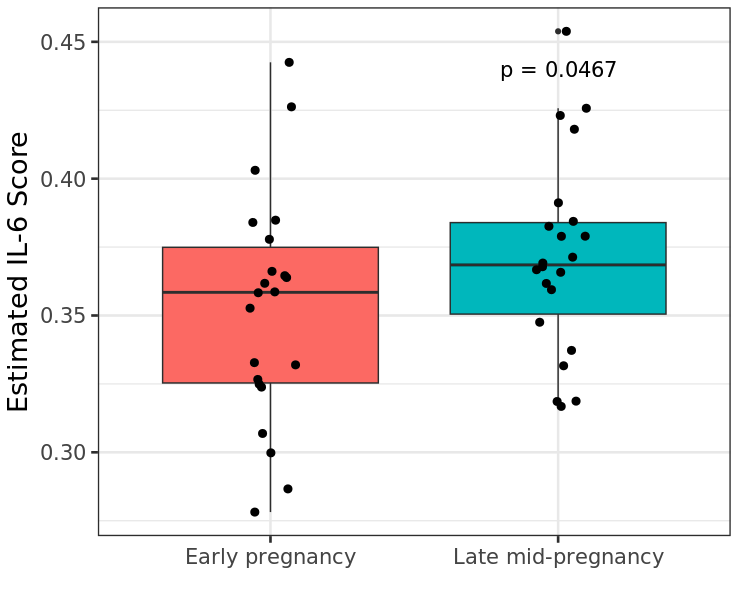


(B)


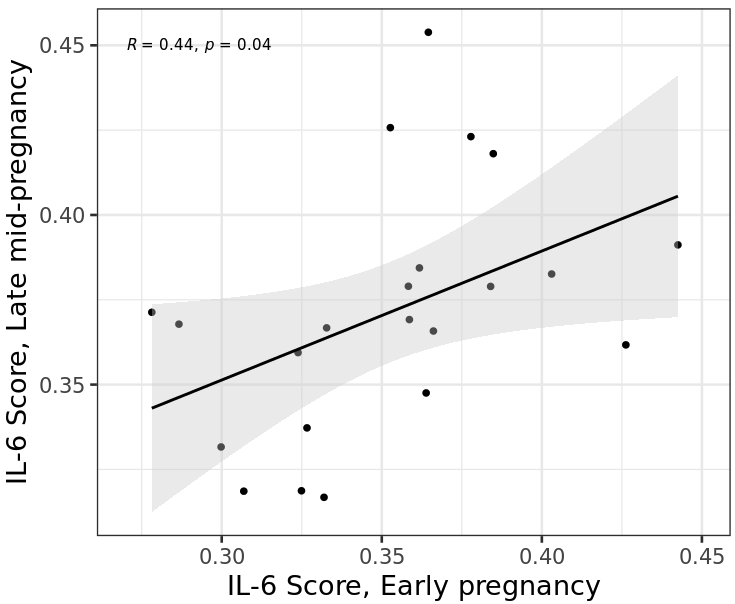


**Supplementary figure 2**: Forest plots displaying the regression coefficients of gestational age specific CpGs (A) discovered at early pregnancy and tested as candidates at late-mid pregnancy, and (B) those discovered at late-mid pregnancy and tested as candidates at early pregnancy. The X-axis represents the regression coefficient and 95% confidence intervals at the discovery (coloured in dark blue) and candidate (coloured in light blue) analyses respectively; the Y-axis represents the gestational age-specific CpGs that were identified at the discovery timepoint and subsequently tested at the other pregnancy timepoint in which it was not previously detected. The CpGs significantly associated with gestational age in the other timepoint as well are highlighted in bold.

(A)


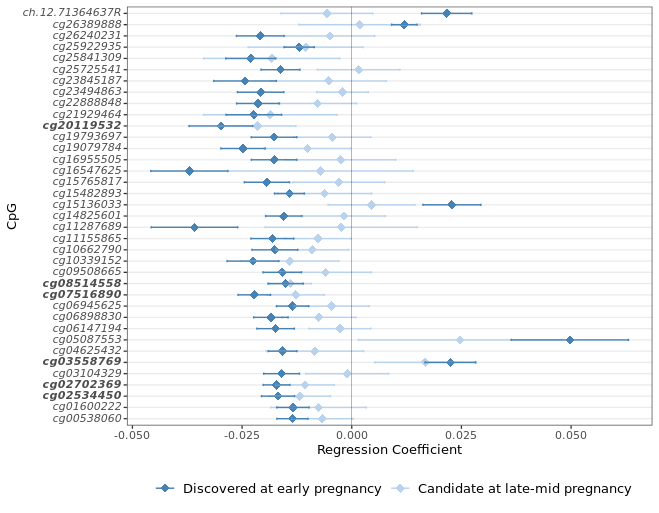


(B)


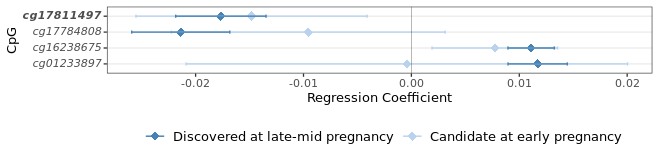


**Supplementary figure 3**: Six CpGs at early pregnancy and 2 CpGs were identified as having a potential SNP-association using the ARIES pregnancy mQTLdb. Bar plots represent the number of pregnancy-related mQTLs at (A) early pregnancy and (B) late-mid pregnancy. The red and blue coloured bars represent cis and trans (with distance greater than, or equal to 1Mb) mQTLs respectively.

(A) (B)


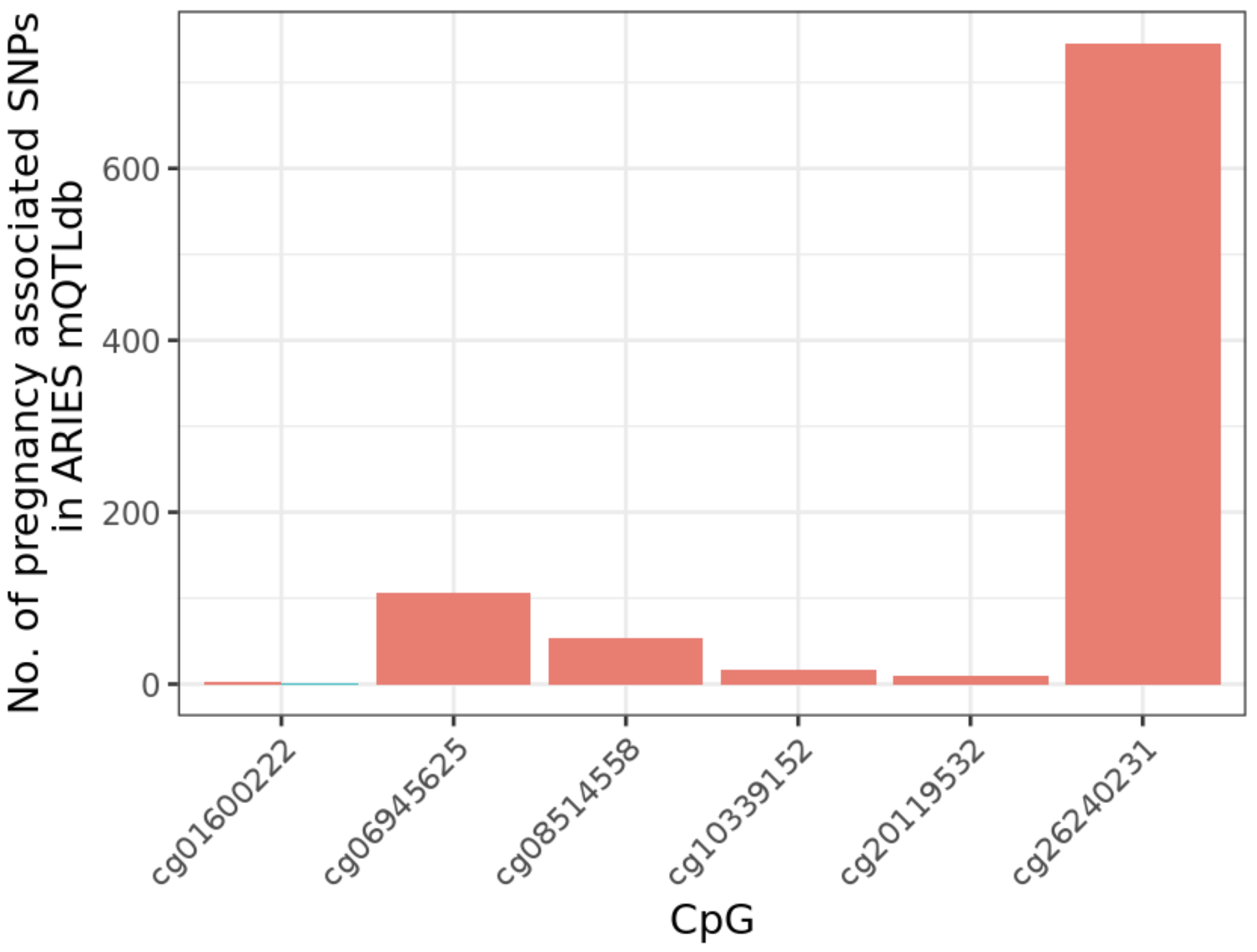

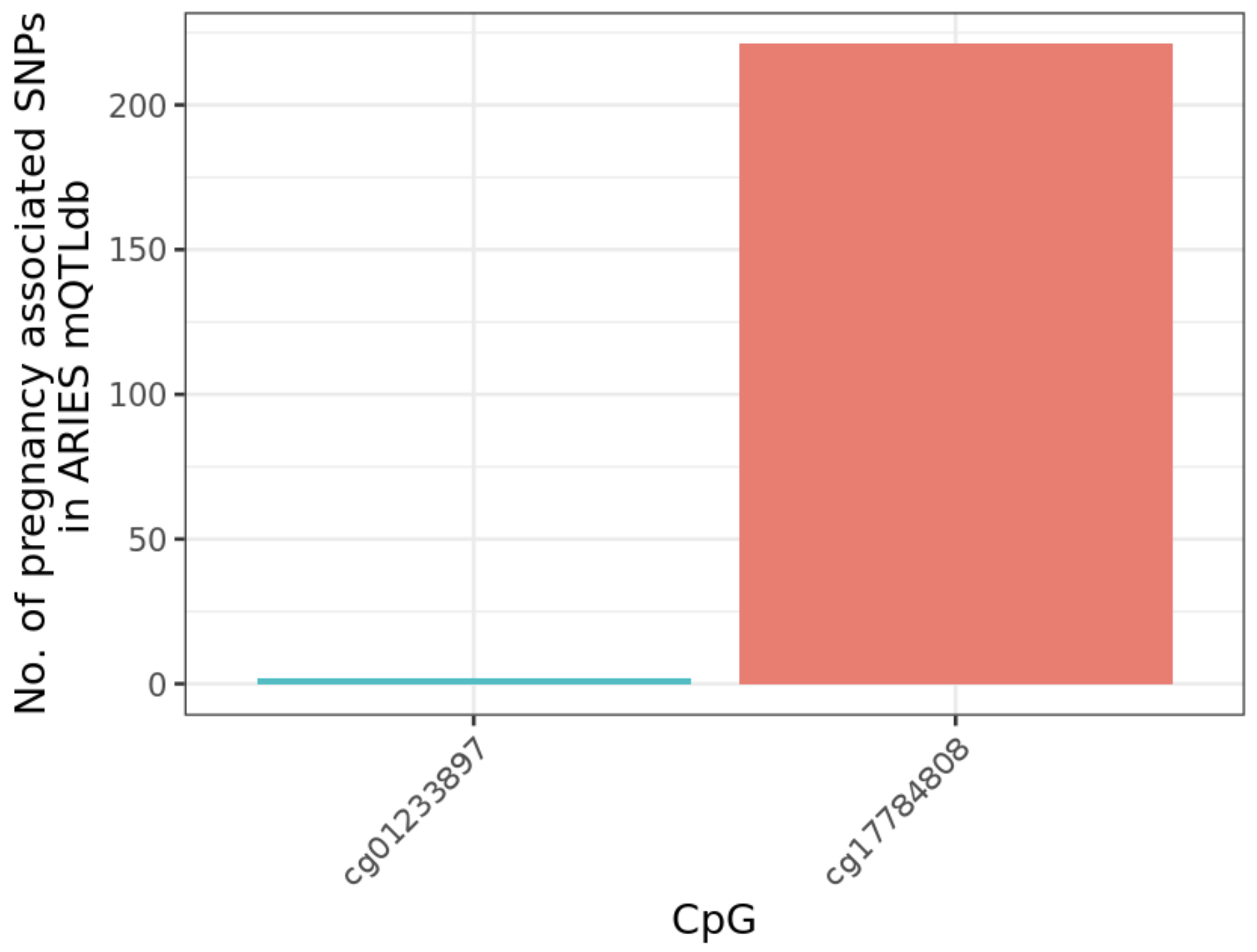


| CpG | Trans-mQTL | Cis-mQTL |
| --- | --- | --- |
| *cg01600222* | 1 | 3 |
| *cg06945625* |  | 107 |
| *cg08514558* |  | 54 |
| *cg10339152* |  | 16 |
| *cg20119532* |  | 10 |
| *cg26240231* |  | 746 |

| CpG | Trans-mQTL | Cis-mQTL |
| --- | --- | --- |
| *cg01233897* | 2 |  |
| *cg17784808* |  | 221 |

**Supplementary figure 4:** Over representation analysis with 18-state ChromHMM annotation identified no significantly enriched chromatin states with the early and late-mid pregnancy timepoint EWAS findings in (A) mononuclear cells or (B) neutrophils. ChromHMM states were obtained from the Roadmap Epigenomics database and overlapped with the EPIC (850K) array annotation to account for background CpGs on the array. Corresponding gene names for the CpGs were identified, and statistical significance for enriched chromatin states was determined at FDR-corrected pvalue <0.1.

1. Mononuclear cells


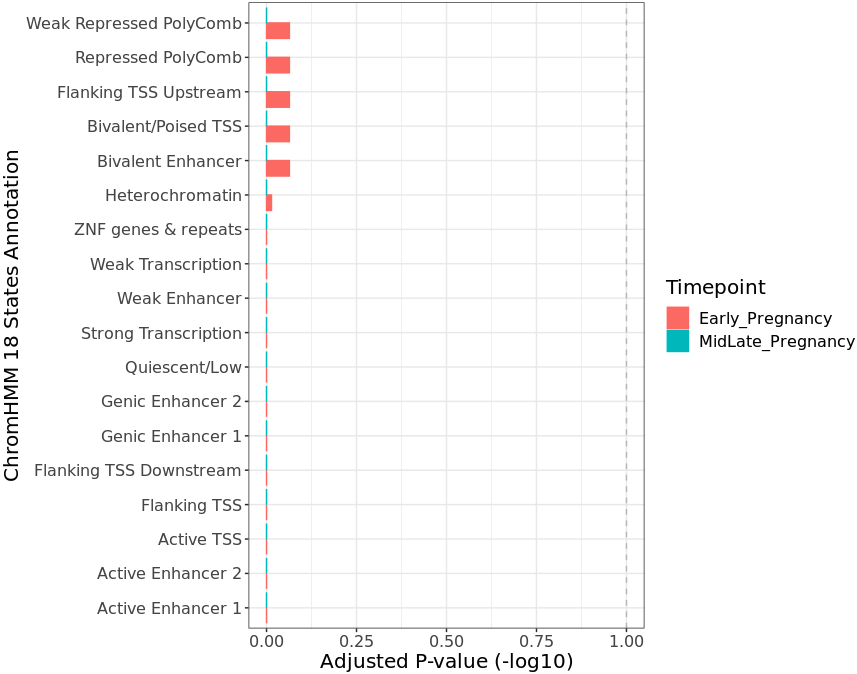


1. Neutrophils


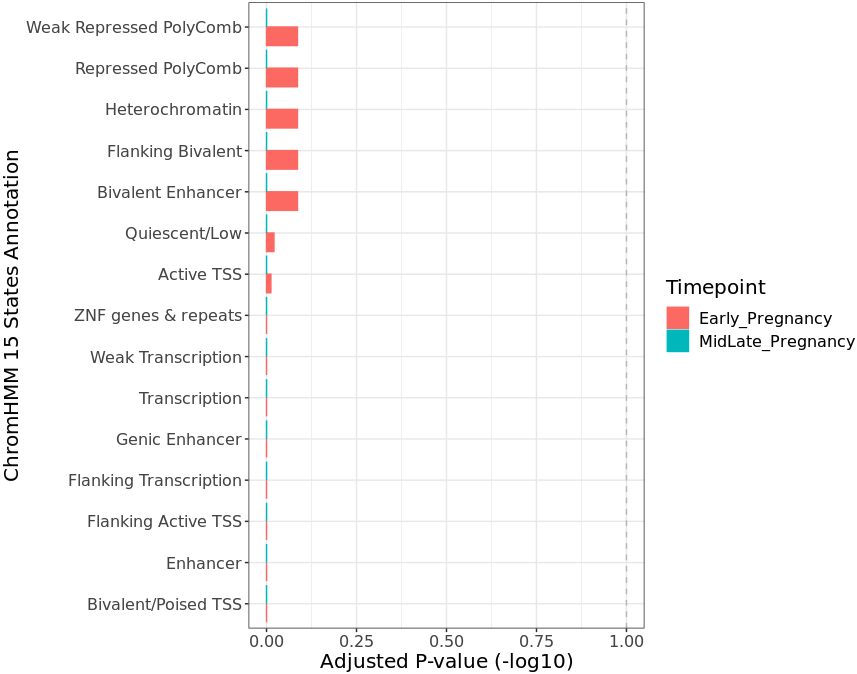


**Supplementary figure 5:** One CpG was significantly different between term and preterm cases at early pregnancy timepoint. (A) Box plot showing significant difference in average beta methylation of *cg06147194* (*FDXR*) between Preterm:Yes (n=4) and Preterm:No/Term (n=22) groups, at early pregnancy. FDR-corrected pvalue was calculated using a non-parametric Wilcoxon test. (B) Pearson correlation of beta methylation of *cg06147194* at early pregnancy with gestational age at birth calculated across all 26 samples. Abbreviation: DB: Δβ, R: Pearson correlation.


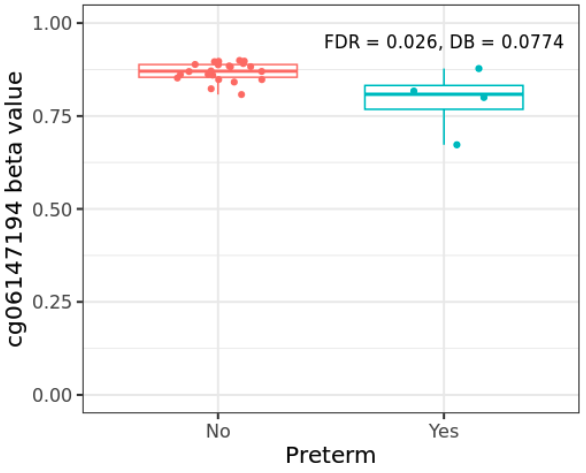


(B)


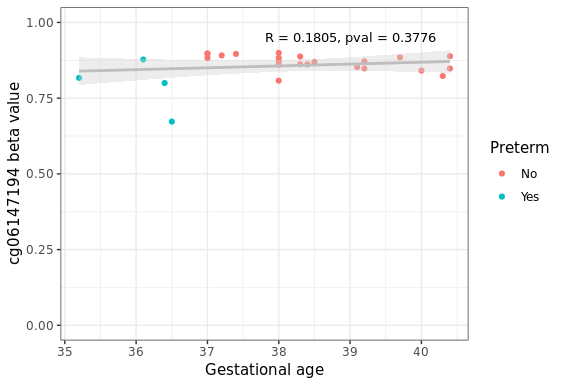

Supplement: Supplementary file 2 — Supplementary Material 2. [file 12884_2025_8037_MOESM2_ESM.docx]
